# Data mining of coronavirus: SARS-CoV-2, SARS-CoV and MERS-CoV

**DOI:** 10.1186/s13104-021-05561-4

**Published:** 2021-04-20

**Authors:** Jung Eun Huh, Seunghee Han, Taeseon Yoon

**Affiliations:** 1 University of Oxford, Oxford, UK; 2 University of Birmingham, Birmingham, UK; 3 Korea University, Seoul, South Korea

**Keywords:** Coronavirus, SARS-CoV-2, SARS-CoV, MERS-CoV, BLAST, Apriori, Decision Tree, SVM

## Abstract

**Objective:**

In this study we compare the amino acid and codon sequence of SARS-CoV-2, SARS-CoV and MERS-CoV using different statistics programs to understand their characteristics. Specifically, we are interested in how differences in the amino acid and codon sequence can lead to different incubation periods and outbreak periods. Our initial question was to compare SARS-CoV-2 to different viruses in the coronavirus family using BLAST program of NCBI and machine learning algorithms.

**Results:**

The result of experiments using BLAST, Apriori and Decision Tree has shown that SARS-CoV-2 had high similarity with SARS-CoV while having comparably low similarity with MERS-CoV. We decided to compare the codons of SARS-CoV-2 and MERS-CoV to see the difference. Though the viruses are very alike according to BLAST and Apriori experiments, SVM proved that they can be effectively classified using non-linear kernels. Decision Tree experiment proved several remarkable properties of SARS-CoV-2 amino acid sequence that cannot be found in MERS-CoV amino acid sequence. The consequential purpose of this paper is to minimize the damage on humanity from SARS-CoV-2. Hence, further studies can be focused on the comparison of SARS-CoV-2 virus with other viruses that also can be transmitted during latent periods.

## Introduction

In this study we compare amino acid and codon sequence of SARS-CoV-2, SARS-CoV and MERS-CoV using different statistics programs to understand how differences in the amino acid and codon sequence can lead to different presentation of the viruses that belong in the same Coronaviridae family. We hypothesize that SARS-CoV and MERS-CoV will have statistically significant amino acid sequence difference to SARS-CoV-2, considering different characteristics of SARS-CoV-2 as seen in Table 1. We hope to identify the main amino acids contributing to this. With this research, we also aim to provide insight on the solution of the current pandemic and suggest future research directions [1–4].

**Table 1:** Materials

	SARS-CoV-2	SARS-CoV	MERS-CoV
Microbiology	Enveloped RNA virus	Enveloped RNA virus	Enveloped RNA virus
Outbreak period	2019-present	2002–2003	2012-present
Initial Site of isolation	Wuhan, China	Guangdong province, China	Saudi Arabia
Countries	214	29	27
No. of cases (mortality)	1,033,187 (2.9%)	8096 (9.6%)	2494 (~ 34%)
Reservoir (intermediate host)	Likely bats (pangolins)	Bats (palm civet)	Bats (dromedary camels)
Incubation period	2–5 days (range, 2–14 days)	2–7 days (range, 2–21)	2–7 (range, 2–14 days)
Infectivity, R0	2.5–3	2.2–3.7 (range, 0.3–4.1)	0.3–1.3
Super spreaders	Yes	Yes	Yes (Uncommon)
Transmission (including to HCP)	Droplet/direct, Airborne/Indirect	Droplet/direct, airborne/indirect?	Droplet/direct, airborne/indirect?
Treatment (PEP)	Dexamethasone, Remdesivir	Supportive (none)	Supportive (None)
Infection prevention	Droplet, contact, face shield	Droplet, contact, face shield	Droplet, contact, face shield

## Main text

### Materials

SARS-CoV-2, SARS-CoV and MERS-CoV share many microbiological similarities. Table 1 visually shows some of the similarities and differences among the viruses.

### Methods

#### Window

Window is a region of a regularly divided peptide sequence. Appropriate window size is important to eliminate variability and to ensure reliable patterns [1–3].

#### FASTA format

FASTA format converts nucleotide sequences or peptide sequences in a single letter code. This allows nucleotide information to be directly inserted into text processing tools [1–3].

#### BLAST

BLAST is provided by NCBI and is used to compare the biological sequence information. Among several different BLAST programs, we chose Nucleotide-nucleotide BLAST(blastn), which finds DNA sequences that are mostly similar to the query DNA from NCBI DNA database.

#### Apriori algorithm

Apriori finds the frequency of individual items in given databases and identifies the relationships among them. In this paper, the itemset consists of different amino acids, which is analyzed by unsupervised Apriori model. Weka program was used [1–3].

#### SVM

SVM is a machine learning algorithm originally based on Statistical Learning Theory. In this paper the supervised SVM model is trained to classify amino acid sequence samples into categories. The aim is to observe the accuracy of the classification models with respect to different hyperplanes. High accuracy model implies the existence of meaningful differences between viruses and allows us to choose adequate hyperplane for classification. SVM-Light library was used [1–3].

#### Decision tree

Decision Tree is a machine learning algorithm that displays decisions and their possible consequences. We used supervised Decision Tree model to classify the cases by training it with given sample to design the questions at each node starting from the root. This allows the case to reach an adequate leaf after satisfying all the conditions of the path. See5 program was used [1–3].

#### Experiment design

We conducted data analysis on the amino acid sequence of SARS-CoV-2, SARS-CoV and MERS-CoV using three methods: BLAST, Apriori and Decision Tree. After interpreting the results of these experiments, we concluded that MERS-CoV is remarkably different from SARS-CoV-2 and SARS-CoV. We conducted further analysis using BLAST, Apriori, SVM and Decision Tree to compare SARS-CoV-2 and MERS-CoV. We compared the codon sequences of the virus to yield more accurate and useful result.

### Result of experiment 1: SARS-CoV-2, SARS-CoV and MERS-CoV

#### BLAST

The result showed that SARS-CoV-2 is almost identical to SARS-CoV while MERS-CoV showed substantial difference. We have experimented the virus with BLAST in pairs. SARS-CoV-2 and SARS-CoV showed 92% identities, 96% positives and 0% gaps which indicates high similarity. SARS-CoV-2 and MERS-CoV showed 51% identities, 66% positives and 3% gaps which indicates relatively low similarity. SARS-CoV and MERS-CoV shows 56% identities, 72% positives and 1% gaps.

#### Apriori

We used Apriori algorithm in 9, 13, 19windows. For each window, we set the minimum support as 0.1, so that associations of more than 10% to the whole instances are regarded as best rules. We defined the rule as the tendency of amino acid A to appear in position N of window, written as posN = A. For accurate analysis, we set the minimum metric confidence level as 0.9 and performed the experiment for 18 cycles.

*9window* Most rules involved Leucine in position 5 with large instances in all three genomes. Additionally, in MERS-CoV, Valine appeared frequently in position 4 and 6.

*13window* All three involved Valine in position 1 and Leucine in position 2 with large instances in both genomes. Additionally, in MERS-CoV, Valine appeared frequently in position 2.

*19window* All three genomes showed Leucine with large instances in some positions. Both SARS-CoV-2 and MERS-CoV involved Valine. In SARS-CoV-2, Valine appears frequently in position 4 and in MERS-CoV, Valine is more dominant than Leucine, appearing frequently in position 4, 6, 9, 11, and 13. SARS-CoV only had one best rule—Leucine in position 1.

These results suggest that Leucine is a commonly significant amino acid in the entire genome for all three viruses. Additionally, the experiments suggest that Valine is a commonly essential amino acid in SARS-CoV-2 and MERS-CoV, especially in MERS-CoV.

##### Decision tree

We defined SARS-CoV-2 as class 1, SARS-CoV as class 2 and MERS-CoV as class 3. We compared the data from the start codon to the stop codon. The characteristics written down are rules of probability over 0.800. This value is high enough to conclude that the species possess a distinguishable trait to the default class. The results are shown in Table 2.

**Table 2:** Decision tree for three viruses

	Species	Rule (default class 1)	Rule (default class 2)	Rule (default class 3)
9window	SARS-CoV-2	pos2 = D & pos9 = Q	pos2 = K & pos7 = N	
	SARS-CoV			
	MERS-CoV	pos3 = W & pos6 = V	pos1 = M & pos4 = F	
		pos3 = S & pos9 = L	pos1 = P & pos7 = T	
		pos2 = F & pos6 = C	pos5 = G & pos7 = M	
		pos3 = I & pos6 = V	pos4 = H & pos7 = N	
		pos2 = L & pos6 = R	pos4 = D & pos7 = _	
		pos1 = Q & pos2 = G	pos5 = E & pos7 = M	
			pos2 = L & pos6 = R	
			pos2 = E & pos4 = N	
			pos2 = L & pos4 = Y	
			pos1 = Y &pos4 = F	
			pos1 = P & pos4 = K	
13window	SARS-CoV-2		pos12 = D & pos13 = N	
	SARS-CoV	pos1 = D & pos13 = V	pos12 = S & pos13 = N	
	MERS-CoV	pos1 = D & pos10 = I	pos11 = H & pos13 = V	
		pos11 = I & pos13 = V	pos5 = V & pos13 = P	
		pos12 L & pos13 = I	pos6 = L & pos13 = G	
		pos12 = A & pos13 = P	pos11 = I & pos13 = E	
		pos12 = V & pos13 = P	pos11 = I & pos13 = V	
		pos6 = I & pos13 = _		
		pos7 = Y & pos13 = A		
		pos11 = V & pos13 = L		
		pos3 = A & pos13 = Q		
		pos11 = F & pos 13 = V		
		pos11 = I & pos13 = V		
		pos5 = V & pos13 = P		
		pos11 = H & pos13 = V		
19window	SARS-CoV-2	pos10 = T & pos12 = K		
	SARS-CoV	pos5 = L & pos10 = V		
		pos4 = I & pos7 = K		
		pos10 = I & pos13 = K		
	MERS-CoV	pos5 = Y & pos10 = V		pos13 = V &pos15 = K
		pos4 = L & pos7 = K		
		pos7 = A & pos12 = Y		
		pos7 = I & pos19 = T		
		pos15 = G & pos16 = I		
		pos13 = L & pos15 = K		
		pos 13 = V & pos15 = K		
		pos15 = V & pos16 = A		
		pos15 = V & pos16 = P		
		pos3 = S & pos15 = G		
		pos12 = E & pos16 = G		
		pos3 = S & pos6 = S		
		pos7 = H & pos11 = I		
		pos2 = S & pos15 = Q		
		pos2 = E & pos15 = Q		
		pos4 = T & pos10 = I		
		pos3 = L & pos10 = L		
		pos7 = S & pos15 = T		
		pos15 = V & pos16 = S		
		pos15 = V & pos16 = V		

*9window* SARS-CoV-2 and MERS-CoV have their unique characteristics that can distinguish them from SARS-CoV-2 and SARS-CoV. However, there weren’t any unique characteristics that can differentiate them from MERS-CoV. SARS-CoV does not have distinct amino acid characteristics compared to the other two viruses. The results show that there are few unique characteristics to distinguish between SARS-CoV-2 and MERS-CoV but that SARS-CoV are more similar to the other two viruses. Also, the results showed that there were no unique characteristics to distinguish the three viruses from MERS-CoV. This means that all three viruses are similar to MERS-CoV.

*13window* SARS-CoV-2 has one unique characteristic to distinguish from default 2. SARS-CoV has one distinct characteristic each to SARS-CoV-2 and SARS-CoV. MERS-CoV has few unique characteristics that can distinguish them from SARS-CoV-2 and SARS-CoV. The results show that there are no unique characteristics to distinguish the three viruses from MERS-CoV. This means that all three viruses are similar to MERS-CoV.

*19window* SARS-CoV-2 has one unique characteristic that can distinguish it from SARS-CoV. SARS-CoV has three distinct characteristics to SARS-CoV-2. MERS-CoV has few unique characteristics that can distinguish them from SARS-CoV-2 and one unique characteristic to SARS-CoV. The results show that there are no unique characteristics to distinguish the three viruses from SARS-CoV. This means that all three viruses are similar to SARS-CoV.

The precision, recall and F-Measure value of the three Decision Tree models we used were all around 0.3. This implies that the models are not reliable to draw accurate results. This is inevitable and was expected as MERS-CoV sequence is very different from that of other two viruses.

### Result of experiment 2: SARS-CoV-2 and MERS-CoV

#### Blast

BLASTN is used to analyze the identicality of SARS-CoV-2 and MERS-CoV. The result shows 59% identity and the distribution of top 8 blast hits on the subject sequence was visible.

Therefore, using the remaining three methods, we compared the two DNA sequences and found appreciable similarities and differences. Throughout following experiments, we chose to compare orf1ab, the first and the longest ORF, of SARS-CoV-2 and MERS-CoV which showed the most remarkable difference between two viruses among several ORFs with the same position.

#### Apriori algorithm

We analyzed the genome of SARS-CoV-2 and MERS-CoV using the Apriori algorithm in 9, 13, 19 windows. Other settings were identical to the previous experiment.

*9window* Most rules involved Leucine in most positions with large instances in both genomes. Additionally, in MERS-CoV, Valine appeared frequently in position 1, 3, 4, and 8.

*13window* Most rules involved Leucine in almost all positions with large instances in both genomes. Additionally, in SARS-CoV-2, Valine appeared frequently in position 4. Also, in MERS-CoV, Valine appeared frequently in position 3, 6, 7, and 13.

*19window *Most rules involve Leucine in almost all positions with large instances in both genomes. Additionally, in SARS-CoV-2, Valine appeared frequently in position 12 and 16; and Threonine also appeared frequently in position 17. Also, in MERS-CoV, Valine appeared frequently in position 2, 13, 14, and 16; Threonine appeared frequently in position 13; and Serine also appeared frequently in position 19.

These results suggest that Leucine is a significant amino acid in both entire genomes. Valine and Threonine are also essential amino acids in certain positions of both genomes, with MERS-CoV having more Valine and Serine.

#### SVM

The result of Apriori experiment suggests that the DNA sequences of the two viruses are very similar, having Leucine as their main amino acid. However, the slight difference such as frequency of Valine and Threonine is not neglectable. SVM algorithm is thus used to validate the significance of the differences found. The SVM experiment is conducted in 9window, 13window, and 19window with four types of functions: normal, polynomial, RBF, and sigmoid. The experiment method was tenfold cross validation.

Normal SVM model have average accuracy slightly over 50%. This low accuracy implies that the differences between the viruses are unidentifiable. Polynomial and sigmoid SVM models showed low accuracy supporting that the viruses are difficult to be differentiated using linear classifying processes.

SVM model of RBF, a non-linear kernel, showed up to 79.94% average accuracy, implying that it is the best chance of classifying the data set. However, the recall value varied from 52.87% to 100%, while the precision varied from 67.09% to 100%. Trained models would have high precision with low recall or high recall with low precision. This indicates that the model was trained to have extremely specialized or general classifying net. We could train several models to have 100% recall as well as precision of 70–75%. Thus, we concluded that the SVM model using RBF kernel is statistically significant enough to prove the existence of meaningful feature that distinguishes the amino acid sequences.

##### Decision tree

We defined SARS-CoV-2 as class 1 and MERS-CoV as class 2. Rules that had the probability of at least 0.850 were selected as distinguishable trait. Table 3 shows that SARS-CoV-2 and MERS-CoV have their unique characteristics in all 9, 13, and 19 window. The results show that there are many unique characteristics to distinguish the two viruses.

**Table 3:** Decision tree for two viruses

Species	Rules in 9window	Rules in 13window	Rules in 19window
SARS-CoV-2	pos3 = L & pos5 = P	pos1 = T & pos10 = G	pos17 = N & pos19 = L
	pos3 = N & pos8 = I	pos5 = L & pos11 = I	pos14 = K & pos18 = L
	pos1 = G & pos3 = V	pos6 = T & pos11 = A	pos12 = T & pos17 = V
		pos2 = R & pos6 = M	pos17 = H
		pos10 = L & pos12 = I	
MERS-CoV	pos1 = Y & pos3 = V	pos10 = Q & pos13 = L	pos4 = V & pos12 = G
	pos1 = V & pos3 = P	pos3 = A & pos10 = T	pos12 = S & pos17 = V
	pos3 = S & pos9 = V	pos6 = C & pos11 = A	pos17 = L & pos18 = V
	pos1 = M & pos3 = V	pos11 = W	
	pos2 = D & pos3 = L	pos5 = S & pos11 = I	
	pos1 = Y & pos3 = V	pos2 = T & pos13 = I	
	pos2 = L & pos3 = Q	pos5 = V & pos11 = D	
	pos1 = Q & pos3 = V	pos6 = V & pos11 = A	
		pos2 = Y & pos4 = S	

## Discussion and conclusion

Comparing the three viruses, the result of BLAST showed that SARS-CoV-2 and SARS-CoV has remarkable difference to MERS-CoV. Apriori experiment specifies that SARS-CoV-2 and SARS-CoV have almost the same distribution of amino acids, having Leucine as their main amino acid. In Decision tree experiment, all three viruses are similar to MERS-CoV in 9 and 11window. The three viruses are similar to SARS-CoV in 19window.

These experiments showed high similarity as well as remarkable difference between SARS-CoV-2 and MERS-CoV, which has led us to conduct further experiments. The result of BLAST showed 59% similarity. The Apriori experiment specified that the viruses are similar in having Leucine and Valine as their main amino acid, and Threonine frequently appearing. However, SVM result showed that though the viruses are very alike, they can be effectively classified using non-linear kernels such as RBF. Decision Tree experiment proved several remarkable properties of SARS-CoV-2 amino acid sequence that cannot be found in MERS-CoV amino acid sequence.

Our experiment results are consistent with the high manifestation resemblance between SARS-CoV-2 and SARS-CoV such as high infectivity, while relatively different presentation in MERS-CoV which has high mortality and low infectivity [4]. However, it was still possible to distinguish between SARS-CoV-2 and SARS-CoV using RBF non-linear kernels, which could possibly explain SARS-CoV-2′s characteristic of infectivity during incubation period.

## Limitations

Decision Tree experiments revealed specific differences and similarities among the viruses. However, as explained above, the precision, recall and F-measure values are low due to significant differences in the amino acid sequence. We therefore suggest further research on this using more accurate algorithm based on our findings.

Viral proteins mutate frequently, which can lead to changes in viral amino acid sequence. This can potentially develop into different presentation of the disease. Our experiment was conducted using the current strain of SARS-CoV-2. This means our results may not be applicable for the different mutations that have been reported around the world. Further research with mutated strains of SARS-CoV-2 is necessary to confirm this.

## Data Availability

The datasets used and/or analysed during the current study are available from the corresponding author on reasonable request.

## Abbreviations

SARS-CoV-2: Severe acute respiratory syndrome coronavirus 2
SARS-CoV: Severe acute respiratory syndrome coronavirus
MERS-CoV: Middle East respiratory syndrome coronavirus
BLAST: Basic Local Alignment Search Tool
SVM: Support Vector Machine
